# Combination of platelet count and mean platelet volume (COP-MPV) predicts postoperative prognosis in both resectable early and advanced stage esophageal squamous cell cancer patients

**DOI:** 10.1007/s13277-015-4774-3

**Published:** 2016-01-16

**Authors:** Fan Zhang, Zhaoli Chen, Pan Wang, Xueda Hu, Yibo Gao, Jie He

**Affiliations:** National Cancer Center, Cancer Hospital and Institute, Chinese Academy of Medical Sciences, Panjiayuannanli 17, Chaoyang District, Beijing, 100021 China

**Keywords:** Esophageal squamous cell cancer, Mean platelet volume, Platelet count, Scoring system, Prognosis

## Abstract

The aim of this study is to search the most powerful prognostic factor from routine blood test for esophageal squamous cell cancer (ESCC) patients. Multiple laboratory tests were evaluated including those reflecting red blood cell parameters (hemoglobin (Hb), mean corpuscular volume (MCV), mean corpuscular hemoglobin concentration (MCHC), and red blood cell distribution width (RDW)), platelet morphological parameters (mean platelet volume (MPV) and platelet count (PLT)), blood coagulation status (D-dimer), and tumor biomarker (CA19-9). Known inflammatory indices (NLR and PLR) were also calculated. A total of 468 patients who were diagnosed with ESCC between December 2005 and December 2008 were retrospectively analyzed in this study. By utilizing univariate and multivariate Cox proportional hazard analyses, we found that PLT and MPV were significantly associated with overall survival (OS) and disease-free survival (DFS) of ESCC patients, with optimal cutoff values of 212 and 10.6, respectively. Moreover, the combination of the preoperative PLT and MPV (COP-MPV) was calculated as follows: patients with both PLT (≥212 × 10^9^ L^−1^) and MPV (≥10.6 fL) elevation were assigned a score of 2, and patients with one or neither were assigned a score of 1 and 0. The COP-MPV was an independent prognostic factor for OS (hazard ratio (HR) 0.378, 95 % confidence interval (CI) 0.241 to 0.593, *P* < 0.001, 0/2) and DFS (HR 0.341, 95 % CI 0.218 to 0.534, *P* < 0.001, 0/2) in multivariate analyses. In subgroup analyses for early (stages I and II) and locally (stage III) advanced stage patients, COP-MPV was found significantly associated with OS and DFS in each group (*P* = 0.025 and *P* = 0.018 for OS and *P* = 0.029 and *P* = 0.002 for DFS). In conclusion, we considered that COP-MPV is a promising predictor for postoperative survival in ESCC patients.

## Introduction

Among the most lethal malignancies, esophageal cancer (EC) ranks the sixth worldwide, leading to approximately 400,000 deaths in 2012 [1]. In China, esophageal cancer is the fourth leading cause of all cancer deaths, representing a major problem of public health in some high-risk rural areas [2]. The dominant histopathological type of EC is squamous cell carcinoma (ESCC) in Chinese patients, which covers 90 % of all cases [3]. Despite the progress in radical resection and adjuvant therapy (radiation and chemotherapy), ESCC still shows a poor 5-year survival rate of less than 30 % [4]. The tumor node metastasis (TNM) staging, especially the status of lymph node metastasis, is currently the best predictor for ESCC patient survival [5]. Although several studies had identified certain survival-related biomarkers [6–8], they were less powerful and hardly able to be converted to clinical use. Thus, it is important to recognize effective and easy-obtained biomarkers for ESCC prognosis.

In the past decade, platelet activation has been demonstrated as a crucial biological process in carcinogenesis and metastasis [9–11]. Platelet count (PLT) and mean platelet volume (MPV) are two main characteristics to evaluate platelet activation [12]. Patients with thrombocytosis have been reported to have worse prognosis in multiple solid tumors, such as ovarian cancer [13], endometrial cancer [14], gastric cancer [15], and colorectal cancer [16]. On the other hand, high MPV reflected an abnormal rate of platelet production and stimulation [17]. Recent studies revealed that MPV levels were relatively higher in tumor patients than in normal controls and associated with poor prognosis in some gastrointestinal neoplasms [18–20]. However, the predictive value of PLT combined with MPV for postsurgery survival in ESCC has not been yet investigated.

In this study, we assessed and checked the prognostic value of preoperative PLT and MPV in 468 ESCC patients. Moreover, we took the utility of a novel prognostic system based on platelet activation, termed combination of platelet count and MPV (COP-MPV), which made a promising distinction between better and worse prognosis in both subgroups of early (stages I and II) and locally advanced stage (stage III) ESCC patients.

## Materials and methods

### Patients

Patients with histopathologically confirmed ESCC with no distal metastasis (TNM stage I–III) were enrolled in the study from December 2005 to December 2008. All patients underwent esophagectomy at the Cancer Institute and Hospital, Chinese Academy of Medical Sciences (CAMS), with written informed consent. The exclusion criteria were (1) severe complications or deaths occurred within 30 days after surgery, (2) preoperative systemic inflammatory response syndrome (SIRS), (3) neoadjuvant radiotherapy or chemotherapy, and (4) evidence of infection or autoimmune disease. All patients underwent a careful preoperative evaluation, including clinical history taking, physical examination, laboratory blood testing (biochemistry, complete blood cell counts, coagulation status, and serum tumor marker), pulmonary function test, and multiple radiography (computed tomography (CT) or magnetic resonance imaging (MRI)). Clinicopathological information of the patients were obtained from the medical records, including age, gender, smoking history, drinking status, tumor location, differentiation grade, maximum tumor diameter, lymph node metastasis, TNM stage, and history of adjuvant chemotherapy and radiotherapy. The TNM stage was assessed according to American Joint Committee on Cancer (AJCC) staging manual (seventh edition) [21]. Patients underwent adjuvant radio/chemotherapy after surgery according to TNM stages and family economic status. The laboratory characteristics, including hemoglobin (Hb), mean corpuscular volume (MCV), mean corpuscular hemoglobin concentration (MCHC), red blood cell distribution width (RDW), mean platelet volume (MPV), platelet count (PLT), D-dimer, and CA19-9, were performed within 5 days prior to surgery. The last follow-up date was July 9, 2015. This study was approved by the medical ethics committee of the Cancer Institute and Hospital, CAMS.

### Statistical analysis

Overall survival (OS) was defined as follows: the time from surgery to the time of patients’ death for any cause or the last follow-up date when the patient was known alive. Disease-free survival (DFS) was calculated from the date of operation to first tumor recurrence. Neutrophil-lymphocyte ratio (NLR)/platelet-lymphocyte ratio (PLR) was, respectively, defined as absolute neutrophil/lymphocyte count divided by absolute lymphocyte count. Categorical variables were shown as frequency (percentage), while continuous variables were presented as the mean values ± standard deviation. Unpaired *t* or *χ* ^2^ test was used to compare whether statistical differences between groups were significant. The optimal cutoff values of all continuous variables were determined by receiver operating characteristic (ROC) curve. We used univariate analysis to narrow down the list of possible prognostic factors. Variables with *P* value < 0.1 in univariate analysis were brought into multivariate Cox proportional hazard model to determine their independency. Specifically, the cutoff values of PLT and MPV were 212 (×10^9^ L^−1^) and 10.6 (fL), respectively. The COP-MPV score was calculated on the basis of these two platelet characteristics. Patients with both a higher platelet count (≥212 × 10^9^ L^−1^) level and a higher mean platelet volume (≥10.6 fL) level were grouped a score of 2, and patients with one or neither were grouped a score of 1 and 0, respectively. Kaplan-Meier curves and log-rank test were used to compare survival differences among groups. All statistical analyses were conducted by SPSS 21.0 software (IBM Corporation, Somers, NY, USA). *P* < 0.05 was considered statistically significant.

## Results

A total of 468 patients were enrolled in this study, including 376 men and 92 women. The mean age of all patients was 59.5 ± 9.0 years (median age 60 years; range 36 to 81). Mean follow-up period was 49.1 ± 32.6 months (range 3.2 to 114.5 months). Two-hundred seventy (57.7 %) patients had died during the observation period. The 5-year overall survival rate was 45.0 % for the whole cohort. The distribution of TNM stages was stage I, 46 (9.8 %); stage II, 199 (42.6 %); and stage III, 223 (47.6 %). Mean PLT and MPV were 218 ± 65 × 10^9^ L^−1^ (range 52 to 611) and 10.6 ± 1.1 fL (range 7.1 to 14.4), respectively. Mean (SD, range) levels of other selected laboratory variables or ratios were as follows: Hb, 145 gL^−1^ (15, 65 to 194); MCV, 94.4 fL (5.1, 76.7 to 114.4); MCHC, 342 gL^−1^ (11, 311 to 378); RDW, 12.8 % (0.8, 10.4 to 16.5); D-dimer, 155 μg L^−1^ (129, 18 to 1522); CA19-9, 11.63 U mL^−1^ (13.83, 0.35 to 145.30); NLR, 1.98 (1.02, 0.53 to 11.00); and PLR, 109.66 (40.20, 32.79 to 418.52).

The relationship between COP-MPV and clinical characteristics of patients with ESCC was shown in Table 1. Most indices had no significant differences among three groups except for sex (*P* = 0.004), smoking history (*P* = 0.034), tumor location (*P* < 0.001), and survival period (*P* < 0.001).

**Table 1:** Clinical characteristics between different COP-MPV groups

Variables	COP-MPV = 0 *n* (%)	COP-MPV = 1 *n* (%)	COP-MPV = 2 *n* (%)	*P* value
Age (year)				0.299
<60	35 (41.2)	160 (50.6)	32 (47.8)	
≥60	50 (58.8)	156 (49.4)	35 (52.2)	
Sex				0.004
Female	20 (23.5)	50 (15.8)	22 (32.8)	
Male	65 (76.5)	266 (84.2)	45 (67.2)	
Smoking				0.034
Ever	57 (67.1)	163 (51.6)	39 (58.2)	
Never	28 (32.9)	153 (48.4)	28 (41.8)	
Drinking				0.157
Yes	36 (42.4)	114 (36.1)	32 (47.8)	
No	49 (57.6)	202 (63.9)	35 (52.2)	
Tumor location				<0.001
Upper	16 (18.8)	187 (59.2)	13 (19.4)	
Middle + lower	69 (81.2)	129 (40.8)	54 (80.6)	
Differentiation				0.916
High	20 (23.5)	84 (26.6)	17 (25.4)	
Moderate	41 (48.2)	157 (49.7)	32 (47.8)	
Poor	24 (28.2)	75 (23.7)	18 (26.9)	
Maximum tumor diameter (cm)				0.930
≥7.0	18 (21.2)	73 (23.1)	15 (22.4)	
<7.0	67 (78.8)	243 (76.9)	52 (77.6)	
*N* metastasis				0.508
Yes	37 (43.5)	160 (50.6)	33 (49.3)	
No	48 (56.5)	156 (49.4)	34 (50.7)	
TNM stage				0.096
I	14 (16.5)	28 (8.9)	4 (6.0)	
II	39 (45.9)	133 (42.1)	27 (40.3)	
III	32 (37.6)	155 (49.1)	36 (53.7)	
Adjuvant radio/chemotherapy				0.484
Yes	42 (49.4)	160 (50.6)	39 (58.2)	
No	43 (50.6)	156 (49.4)	28 (41.8)	
Survival period (m)	59.7 ± 30.2	48.7 ± 33.3	37.9 ± 28.1	<0.001

Table 2 showed the distribution of multiple laboratory variables in three groups divided by COP-MPV. MPV (*P* < 0.001), PLT (*P* < 0.001), D-dimer (*P* = 0.007), and PLR (*P* = 0.004) showed significant differences.

**Table 2:** Laboratory characteristics between different COP-MPV groups

Variables	COP-MPV = 0 (*n* = 75)	COP-MPV = 1 (*n* = 316)	COP-MPV = 2 (*n* = 67)	*P* value
Hb (gL^−1^)	144.0 ± 16.3	145.2 ± 14.7	144.1 ± 14.7	0.728
MCV (fL)	94.3 ± 5.2	94.6 ± 5.2	93.3 ± 4.6	0.149
MCHC (gL^−1^)	343.1 ± 10.1	342.8 ± 11.3	339.4 ± 11.5	0.064
RDW (%)	12.7 ± 0.8	12.8 ± 0.7	12.9 ± 0.7	0.203
MPV (fL)	9.8 ± 0.6	10.6 ± 1.1	11.3 ± 0.5	<0.001
PLT (×10^9^ L^−1^)	174.6 ± 28.2	221.9 ± 70.3	254.4 ± 32.9	<0.001
D-dimer (μg L^−1^)	186.3 ± 201.4	142.0 ± 99.1	175.9 ± 129.6	0.007
CA19-9 (U mL^−1^)	10.7 ± 6.9	12.2 ± 16.2	10.2 ± 6.2	0.916
NLR	1.9 ± 1.0	2.0 ± 1.0	1.9 ± 1.4	0.517
PLR	97.0 ± 30.0	111.7 ± 43.4	116.3 ± 32.4	0.004

We next separated the cohort into different groups by cutoff values of clinicolaboratory variables. Survival analyses of OS and DFS in relation to every selected variable were performed. In univariate analyses, age (≥60/<60) (hazard ratio (HR) 1.537, 95 % confidence interval (CI) 1.206 to 1.959, *P* = 0.001), lymph node metastasis (presence/absence) (HR 2.134, 95 % CI 1.671 to 2.726, *P* < 0.001), TNM stage (I + II/III) (HR 0.434, 95 % CI 0.340 to 0.554, *P* < 0.001), adjuvant therapy (yes/no) (HR 1.749, 95 % CI 1.370 to 1.224, *P* < 0.001), smoking (ever/never) (HR 1.297, 95 % CI 1.021 to 1.647, *P* = 0.033), maximum tumor diameter (≥7.0/<7.0 cm) (HR 1.580, 95 % CI 1.206 to 2.070, *P* = 0.001), RDW (≥12.2/<12.2 %) (HR 1.505, 95 % CI 1.068 to 2.122, *P* = 0.020), MPV (≥10.6/<10.6 fL) (HR 1.354, 95 % CI 1.066 to 1.720, *P* = 0.013), PLT (≥212/<212 × 10^9^ L^−1^) (HR 1.332, 95 % CI 1.048 to 1.692, *P* = 0.019), CA19-9 (≥4.79/<4.79 U mL^−1^) (HR 1.561, 95 % CI 1.148 to 2.123, *P* = 0.005), NLR (≥2.50/<2.50) (HR 1.417, 95 % CI 1.058 to 1.899, *P* = 0.019), PLR (≥117.07/<117.07) (HR 1.288, 95 % CI 1.003 to 1.653, *P* = 0.047), and COP-MPV (HR 0.394, 95 % CI 0.254 to 0.611, *P* < 0.001) were associated with OS (Table 3). Similar results were revealed in the relationships of these factors with DFS (Table 3). Multivariate analyses demonstrated that age (HR 1.595, 95 % CI 1.236 to 2.059, *P* < 0.001), lymph node metastasis (HR 1.869, 95 % CI 1.421 to 2.458, *P* < 0.001), adjuvant therapy (HR 1.327, 95 % CI 1.006 to 1.751, *P* = 0.045), maximum tumor diameter (HR 1.365, 95 % CI 1.019 to 1.828, *P* = 0.037), CA19-9 (HR 1.740, 95 % CI 1.270 to 2.385, *P* < 0.001), and COP-MPV (HR 0.378, 95 % CI 1.270 to 2.385, *P* < 0.001) were independent prognostic factors of ESCC patients (Table 4).

**Table 3:** Univariate analyses of overall survival and disease-free survival for all ESCC patients

Variables	*P* value	OS HR (95 % CI)	*P* value	DFS HR (95 % CI)
Age (≥60 vs <60)	0.001	1.537	0.002	1.459
		(1.206 to 1.959)		(1.145 to 1.860)
Sex (male vs female)	0.591	1.088	0.496	1.113
		0.800 to 1.479		0.818 to 1.513
Tumor location (upper vs middle + lower)	0.444	1.143	0.388	1.163
		(0.811 to 1.611)		(0.825 to 1.639)
Differentiation (high, moderate, and poor)	0.910	1.019	0.952	1.010
		(0.738 to 1.407)		(0.732 to 1.395)
*N* metastasis (presence vs absence)	<0.001	2.134	<0.001	2.116
		(1.671 to 2.726)		(1.657 to 2.702)
TNM stage (I + II vs III)	<0.001	0.434	<0.001	0.433
		(0.340 to 0.554)		(0.339 to 0.553)
Adjuvant radio/chemotherapy (yes vs no)	<0.001	1.749	<0.001	1.846
		(1.370 to 2.234)		(1.445 to 2.358)
Smoking (ever vs never)	0.033	1.297	0.025	1.313
		(1.021 to 1.647)		(1.034 to 1.667)
Drinking (yes vs no)	0.576	1.073	0.601	1.068
		(0.839 to 1.372)		(0.835 to 1.366)
Maximum tumor diameter (≥7.0 vs <7.0 cm)	0.001	1.580	0.001	1.599
		(1.206 to 2.070)		(1.220 to 2.095)
Hb (≥135 vs <135 gL^−1^)	0.228	0.847	0.251	0.854
		(0.647 to 1.109)		(0.652 to 1.118)
MCV (≥98.7 vs <98.7 fL)	0.070	1.329	0.071	1.327
		(0.977 to 1.808)		(0.976 to 1.806)
MCHC (≥344 vs <344 gL^−1^)	0.755	1.039	0.660	1.055
		(0.817 to 1.321)		(0.830 to 1.342)
RDW (≥12.2 vs <12.2 %)	0.020	1.505	0.027	1.474
		(1.068 to 2.122)		(1.046 to 2.077)
MPV (≥10.6 vs <10.6 fL)	0.013	1.354	0.015	1.347
		(1.066 to 1.720)		(1.060 to 1.710)
PLT (≥212 vs <212 × 10^9^ L^−1^)	0.019	1.332	0.003	1.431
		(1.048 to 1.692)		(1.127 to 1.819)
D-dimer (≥207 vs <207 μg L^−1^)	0.164	1.215	0.231	1.182
		(0.924 to 1.597)		(0.899 to 1.554)
CA19-9 (≥4.79 vs <4.79 U mL^−1^)	0.005	1.561	0.005	1.559
		(1.148 to 2.123)		(1.147 to 2.120)
NLR (≥2.50 vs <2.50)	0.019	1.417	0.024	1.400
		(1.058 to 1.899)		(1.045 to 1.875)
PLR (≥117.07 vs <117.07)	0.047	1.288	0.039	1.301
		(1.003 to 1.653)		(1.014 to 1.670)
COP-MPV (0, 1, and 2)	<0.001	0.394 (0–2)	<0.001	0.350 (0–2)
		(0.254 to 0.611)		(0.226 to 0.543)
		0.691 (1–2)		0.630 (1–2)
		(0.504 to 0.948)		(0.459 to 0.863)

**Table 4:** Multivariate analyses of overall survival and disease-free survival for all ESCC patients

Variables	*P* value	OS HR (95 % CI)	*P* value	DFS HR (95 % CI)
Age (≥60 vs <60)	<0.001	1.595	0.002	1.498
		(1.236 to 2.059)		(1.162 to 1.930)
*N* metastasis (yes vs no)	<0.001	1.869	<0.001	1.816
		(1.421 to 2.458)		(1.381 to 2.389)
Adjuvant radio/chemotherapy (yes vs no)	0.045	1.327	0.015	1.411
		(1.006 to 1.751)		(1.070 to 1.860)
Smoking (ever vs never)	0.622	1.066	0.611	1.068
		(0.827 to 1.374)		(0.830 to 1.373)
Maximum tumor diameter (≥7.0 vs <7.0 cm)	0.037	1.365	0.046	1.348
		(1.019 to 1.828)		(1.006 to 1.806)
MCV (≥98.7 vs <98.7 fL)	0.095	1.310	0.092	1.311
		(0.954 to 1.798)		(0.957 to 1.796)
RDW (≥12.2 vs <12.2 %)	0.095	1.356	0.101	1.349
		(0.948 to 1.940)		(0.943 to 1.929)
CA19-9 (≥4.79 vs <4.79 U mL^−1^)	0.001	1.740	0.001	1.722
		(1.270 to 2.385)		(1.258 to 2.358)
NLR	0.124	1.283	0.180	1.245
		(0.934 to 1.763)		(0.904 to 1.714)
PLR	0.187	1.203	0.172	1.210
		(0.915 to 1.582)		(0.920 to 1.591)
COP-MPV (0, 1, and 2)	<0.001	0.378 (0–2)	<0.001	0.341 (0–2)
		(0.241 to 0.593)		(0.218 to 0.534)
		0.677 (1–2)		0.620 (1–2)
		(0.490 to 0.535)		(0.449 to 0.856)

Moreover, we examined the prognostic value of COP-MPV in subgroups of early and advanced stages of ESCC patients. Hazard ratios of OS and DFS were found significantly different among three COP-MPV groups in both early (OS, *P* = 0.025; DFS, *P* = 0.018) and advanced stages (OS, *P* = 0.029; DFS, *P* = 0.002). However, PLT or MPV alone did not show that predictive function as they combined (MPV for early stage, *P* = 0.334 (Fig. 2a); MPV for advanced stage, *P* = 0.085 (Fig. 2b); and PLT for advanced stage, *P* = 0.254 (Fig. 3b)) (Table 5).

**Table 5:** Univariate analyses of overall survival and disease-free survival for early stage (TNM I and II) and locally advanced (TNM III) stage patients

Variables	TNM stage	*P* value	OS HR (95 % CI)	*P* value	DFS HR (95 % CI)
MPV (≥10.6 vs <10.6 fL)	I and II	0.334	1.203	0.349	1.196
			(0.827 to 1.752)		(0.822 to 1.742)
	III	0.085	1.317	0.098	1.303
			(0.963 to 1.801)		(0.952 to 1.782)
PLT (≥212 vs <212 × 10^9^ L^−1^)	I and II	0.024	1.545	0.014	1.602
			(1.059 to 2.254)		(1.099 to 2.337)
	III	0.254	1.198	0.050	1.365
			(0.878 to 1.634)		(1.000 to 1.862)
COP-MPV (0, 1, and 2)	I and II	0.025	0.400 (0–2)	0.018	0.385 (0–2)
			(0.204 to 0.786)		(0.196 to 0.756)
			0.732 (1–2)		0.727 (1–2)
			(0.438 to 1.225)		(0.435 to 1.215)
	III	0.029	0.465 (0–2)	0.002	0.371 (0–2)
			(0.259 to 0.833)		(0.207 to 0.665)
			0.671 (1–2)		0.554 (1–2)
			(0.450 to 1.001)		(0.371 to 0.829)

**Fig. 1:**
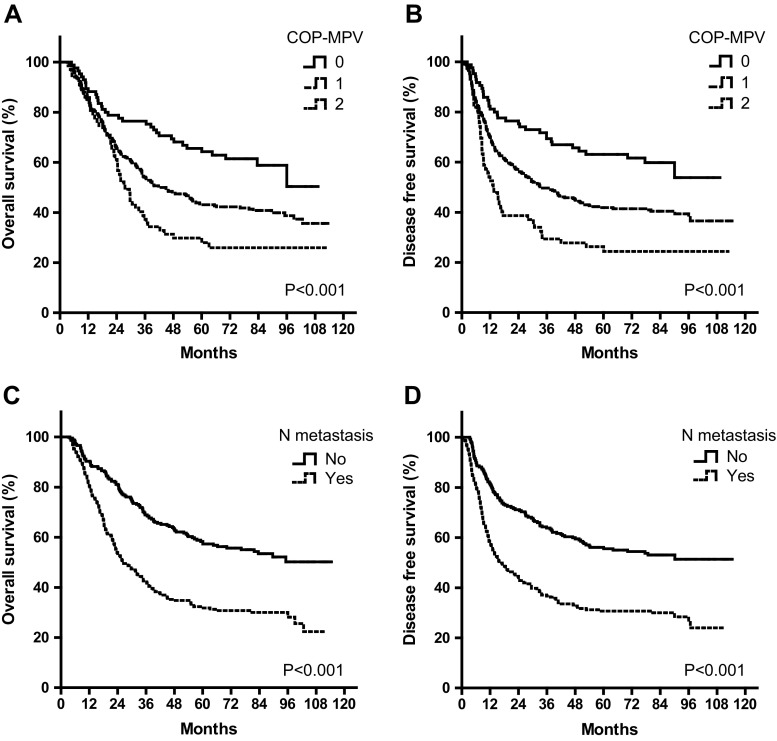
Kaplan-Meier curves of overall survival (**a**) and disease-free survival (**b**) for 468 ESCC patients by COP-MPV category. *P* values were determined by the log-rank test. Relationship between known prognostic factors (lymph node metastasis status) and OS/DFS were shown in (**c**) and (**d**) for comparison

**Fig. 2:**
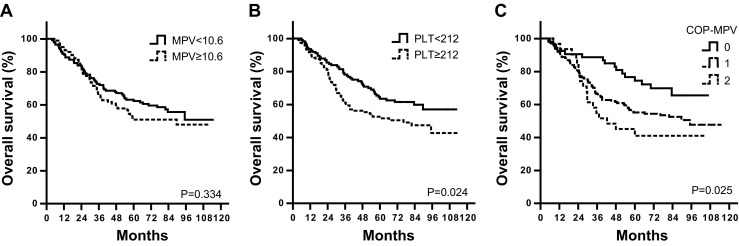
Kaplan-Meier curves of overall survival for stages I and II patients (*n* = 245) by **a** MPV, **b** PLT, and **c** COP-MPV categories. *P* values were determined by the log-rank test. COP-MPV category showed best predictive power

**Fig. 3:**
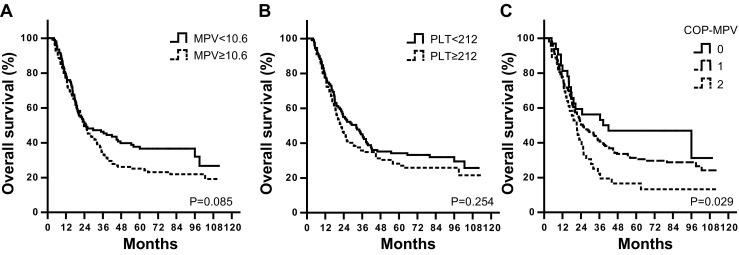
Kaplan-Meier curves of overall survival for stage III patients (*n* = 223) by **a** MPV, **b** PLT, and **c** COP-MPV categories. *P* values were determined by the log-rank test. COP-MPV category showed best predictive power

By Kaplan-Meier analyses, significant differences in OS and DFS among COP-MPV groups were demonstrated (*P* < 0.001), where COP-MPV = 2 group tended to have worse prognosis than other two groups (Fig. 1a, b). The 5-year survival rates for groups 0, 1, and 2 were 64.3, 43.1, and 28.0 %, respectively. We illustrated the effect of one known prognostic factor (lymph node metastasis) to postoperative survival for comparison (Fig. 1c, d). In subgroup analyses, COP-MPV showed its predictive value in both early (TNM stages I and II) and advanced stages (TNM stage III) of ESCC patients. In the early stage subgroup, patients with COP-MPV = 2 were prone to live shorter compared to 0 or 1 groups (*P* = 0.025) (Fig. 2c), where 5-year survival rates for groups 0, 1, and 2 were 74.5, 55.2, and 44.1 %, respectively. Similar results were revealed in the advanced stage group (*P* = 0.029; Fig. 3c), where 5-year survival rates for groups 0, 1, and 2 were 46.9, 31.3, and 16.7 %, respectively.

## Discussion

Platelet activation has been previously reported as a common phenomenon in cardiovascular diseases such as acute ischemic stroke, myocardial infarction, and renal artery stenosis [22, 23]. Recently, more attention has been paid on the clinical significance of this process in several malignancies [24, 25]. The two main aspects to assess the platelet activation status are PLT and MPV [12]. Researchers noticed that many cancers were related to elevation of platelet count in peripheral blood (or thrombocytosis), which were considered to be caused by upstream cytokine stimulation [26] or oncogenesis itself [27, 28]. Emerging evidence suggested that high PLT or PLR indicated poor postoperative survival in several solid tumors [13, 29–31]. On the other hand, MPV also showed promising utility to stratify benign and malignant diseases, although high MPV indicated that malignant disease/poor survival remained controversial [32–34]. Therefore, it is reasonable to combine PLT and MPV as a scoring system (COP-MPV) for platelet activation to evaluate the prognosis of cancer patients.

In this study, we evaluated the association between multiple clinicopathological variables and OS/DFS and found that COP-MPV had the best discriminatory ability as lymph node metastasis status (Table 4 and Fig. 1). NLR and PLR were also significant prognostic factors in univariate analysis but not independent in multivariate Cox regression. This was possibly because their stratifying ability was absorbed by the COP-MPV score. Moreover, we revealed that COP-MPV had a predictive utility in both early and advanced subgroups of ESCC patients (Table 5 and Figs. 2c and 3c). In fact, PLT was more favorable to stratify the survival period for early stage ESCC patients (*P* = 0.024; Fig. 2b), while MPV for advanced stage was though not significant (*P* = 0.085; Fig. 3b). The combination of PLT and MPV was effective in both subgroups as it seemed gathering the advantages of the two indices measuring platelet activation.

The history for investigating MPV as a tumor prognostic marker was not long, but evidence was increasing [32, 35–37]. For instance, Aksoy and colleagues demonstrated that solid tumors with bone marrow metastasis were more likely to have low MPV [32]; a Korean group revealed that high MPV stratified liver cancer compared to normal controls [35]. MPV level was found significantly higher in advanced endometrial cancer compared to early stage patients or healthy controls [36]. Nevertheless, the relationship of MPV value and overall survival was not consistent in different studies. In non-small-cell lung cancer (NSCLC), two Japanese groups announced that low MPV level was associated with unfavorable survival [34, 37]. Their explanation was that the product for PLT × MPV was relatively constant. On the other hand, massive researches proved that high PLT level predicted short postoperative survival [13–16]. Thus, it was reasonable that low MPV level (=high PLT level) resembles active inflammation status which could cause poor prognosis. However, evidence for relationship of high MPV and advanced stage cancer (or unfavorable disease-like thrombotic state) is also emerging, such as in colon cancer [38], blood tumor [39], renal cancer [40], hepatocellular carcinoma [18, 35], gastric cancer [20], and endometrial cancer [36]. The underlying mechanism was also straightforward—the process of platelet activation, stimulated by inflammatory factor such as interleukin-6, was likely to produce platelet with both characteristics, massive and giant, not only one. Therefore, we could expect that both platelet count and volume would be increasing in an unfavorable disease compared to a more benign one. Our study in ESCC supported the latter.

An apparent obstacle for applying these direct blood indices (such as PLT or MPV) is to determine the cutoff values. In our study, 41 (8.8 %) patients were with thrombocytosis (PLT > 300 × 10^9^ L^−1^) and 9 (1.9 %) patients were with abnormal high MPV (>13.0 fL), which had hardly statistically significance on overall survival. However, previous studies on prognostic potential of these markers revealed that poor survival was more likely to be related to elevated tendency, not necessarily to abnormal value (i.e., thrombocytosis or high MPV) [29, 41]. Actually, indirect indices like PLR or NLR were widely used partly because the cutoff values for these indirect indices were not unique. Therefore, our cutoff values for PLT and MPV based on ROC curves, though not the normal-abnormal dividing line, were plausible.

In conclusion, our study proved that combined blood biomarker COP-MPV has prognostic value in 468 ESCC patients. Besides, the predictive ability is effective in both early (TNM I and II) and advanced (TNM III) subgroup patients. To the best of our knowledge, this is the first time to combine the two platelet activation markers together to evaluate their prognostic potential, which would help clinicians to predict the survival of ESCC patients.
